# People confabulate with high confidence when their decisions are supported by weak internal variables

**DOI:** 10.1093/nc/niab004

**Published:** 2021-03-10

**Authors:** Benjamin Rebouillat, Jean Maurice Leonetti, Sid Kouider

**Affiliations:** Brain and Consciousness Group (ENS, CNRS), Département d’Études Cognitives, École Normale Supérieure—PSL Research University, 75005 Paris, France; Ecole Doctorale Cerveau Cognition Comportement, ENS/ParisVI/ParisV, Paris 75005, France; Brain and Consciousness Group (ENS, CNRS), Département d’Études Cognitives, École Normale Supérieure—PSL Research University, 75005 Paris, France; Ecole Doctorale Cerveau Cognition Comportement, ENS/ParisVI/ParisV, Paris 75005, France; Brain and Consciousness Group (ENS, CNRS), Département d’Études Cognitives, École Normale Supérieure—PSL Research University, 75005 Paris, France

**Keywords:** metacognition, agency, intention, volition, contents of consciousness, brain–computer interface

## Abstract

People can introspect on their internal state and report the reasons driving their decisions but choice blindness (CB) experiments suggest that this ability can sometimes be a retrospective illusion. Indeed, when presented with deceptive cues, people justify choices they did not make in the first place, suggesting that external cues largely contribute to introspective processes. Yet, it remains unclear what are the respective contributions of external cues and internal decision variables in forming introspective report. Here, using a brain–computer interface, we show that internal variables continue to be monitored but are less impactful than deceptive external cues during CB episodes. Moreover, we show that deceptive cues overturn the classical relationship between confidence and accuracy: introspective failures are associated with higher confidence than genuine introspective reports. We tracked back the origin of these overconfident confabulations by revealing their prominence when internal decision evidence is weak and variable. Thus, introspection is neither a direct reading of internal variables nor a mere retrospective illusion, but rather reflects the integration of internal decision evidence and external cues, with CB being a special instance where internal evidence is inconsistent.

Highlights

- People’s introspection about their decisions is subject to illusions such as choice blindness.
- A brain–computer interface was used to track people’s choices and manipulate external feedback cues online.
- Introspective reports are influenced both by internal decision variables and external cues.
- Introspective illusions occur when internal decision evidence is unreliable.
- Confident confabulations result from a biased integration in which external cues dominate.

## Introduction

Humans constantly monitor their choices and actions to adapt their behavior (Ericson and Simon 1980; Ridderinkhof *et al.* 2004; Ullsperger and von Cramon 2004; Walton *et al.* 2004). This ability typically involves introspective mechanisms that are used to evaluate and justify decisions (Moshman 2014). Yet, introspection turns out to be unreliable on many occasions (Nisbett and Wilson 1977). For instance, participants can believe they have intentionally performed an action that was actually initiated by another agent (Wegner and Wheatley 1999; Wegner 2017). Similarly, participants can confabulate about why they choose an option while they actually made the opposite choice in the first place (Johansson *et al.* 2005, 2006; Hall *et al.* 2010). A striking example of introspective illusion is given by choice blindness (CB) experiments. In this paradigm, participants select which one of two faces is more attractive, and are then presented with the option they selected and asked to justify their decision. On some trials, they are lured to have chosen the non-preferred face. Yet, they provide confabulated justifications about why this face is more attractive than the other. This phenomenon, which has been extended to economic decisions, political preferences and moral judgments, reveals that introspection can be, under certain circumstances, a retrospective illusion (Hall *et al.* 2012; Hall *et al.* 2013; McLaughlin and Somerville 2013; Strandberg *et al.* 2018).

Yet, participants have also been shown to have reasonable introspective access to the elements driving their decisions (Ericson and Simon 1980; Grover 1982; Schultze-Kraft *et al.* 2016; Reyes *et al.* 2018; Parés-Pujolràs *et al.* 2019). These apparently contradicting results could be reconciliated under an integrative account of introspection where both internal decision variables and external, contextual cues contribute to participants’ introspective reports. Yet, although such view began to receive empirical support (Schultze-Kraft *et al.* 2020), the modalities under which these two components could be integrated during introspective processes remained unsettled.

One way to investigate the formation of introspection about decisions consists in studying how internal decision variables impact CB episodes. In line with previous Bayesian integrative accounts of introspective processes (Moore and Fletcher 2012; Legaspi and Toyoizumi 2019), we predicted that the impact of internal decision evidence on introspection would be mediated by its availability and reliability. Furthermore, we expected that integrative processes would modulate not only the quantity of introspective failures (i.e. the amount of CB episodes) but also their quality (i.e. how much participants are convinced in their confabulation). That is, when a reliable source of external cue sometimes provides a deceptive information, participants would confabulate with high confidence when their internal decision evidence is weak.

**Figure 1.**
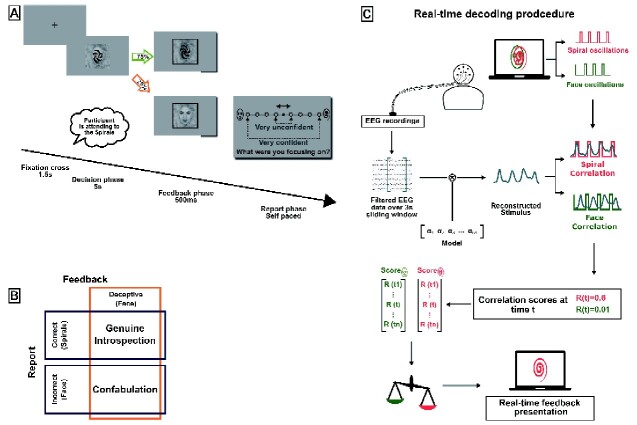
(**A**) Experimental paradigm. Each trial comprised three phases. (i) Decision phase: participants were presented with overlapping face and spiral oscillating at 1.875 Hz in temporal phase opposition and asked to freely focus on one or the other category. (ii) Feedback phase: participants were then presented with a feedback cue for 500 ms, reflecting their recent decision on 75% of the trials (green, informative trial) or the opposite choice in the 25% remaining trials (orange, deceptive trial). (iii) Report phase: participants were then requested to report the object they preferentially attended just before the feedback cue along with their confidence in this report on a four steps scale. (**B**) Relationship between report accuracy and introspective accuracy. After deceptive feedback, a correct report corresponds to a genuine introspection while an incorrect report corresponds to a confabulation. However, in the case of informative trials, reports accuracy does not inform on the nature of introspective mechanisms because participants can be correct just by blindly accepting the feedback. (**C**) Real-time decoding procedure. Each 250 ms, a reconstructed stimulus was computed by linearly combining the 64 EEG electrodes signal over a 3 s window according to the model weights computed beforehand. We then obtain correlation scores for both face (green) and spiral (red) stimuli by computing the correlation between the reconstructed stimulus and the expected face and spiral oscillations respectively. On the end of each trial, correlation scores computed during the last 1.5 s were averaged separately for each category, and the highest average was considered the attended category for the presentation of the feedback cue.

Here, to address this issue, we relied on a brain–computer interface (BCI) setup to track participants’ internal decision variables during the original choice (i.e. prior to the external cue and report). Participants had to freely choose to preferentially attend to one out of two overlapping stimuli while their EEG (electroencephalogram) was recorded and a marker of selective attention was measured in real-time (decision phase, see Fig. 1A). Recent neuroimaging studies revealed that top-down attentional mechanism reflect decision processes (Gottlieb and Balan 2010; Roy *et al.* 2014; Hunt *et al.* 2018). Although we did not measure decision mechanisms *per se*, our neural index allowed us to track their consequence in the form of selectively attending to one category over the other. This BCI setup allowed not only to measure a proxy of internal decision evidence independently of introspective reports but also to control for the reliability of external cues. Following the decision phase, participants were presented with a feedback cue that matched their original choice in 75% of the trials (informative feedback). Importantly, they were presented with the alternative, non-preferred choice as the outcome of their recent decision in 25% of the trials (deceptive feedback). Moreover, in order to assess the impact of internal decision variables not only on the quantity of introspective failures but also on their quality, participants were asked to rate the confidence they experienced in their decision.

## Materials and Methods

### Participants

Thirty healthy participants with normal or corrected-to-normal vision took part in the experiment [14 males; all right-handed; mean age: 25.1 years, standard error to the mean (*SEM*) = 3.4]. Two additional participants were tested but were not included in the analysis because of EEG artifacts (*N* = 1) or technical failures (*N* = 1) altering the online experiment. All participants signed a written consent and received financial compensation in exchange for their participation. This experimental protocol was approved by the local ethical committee (Conseil d'évaluation éthique pour les recherches en santé, Paris, France).

Participants performed 480 trials in the main experiment. All trials contained a decision and feedback phase. The feedback was informative in 75% of the trials, but deceptive in the remaining 25%. Introspective reports were required on all deceptive trials, but only on a third of the informative trials, in order to balance them across cue validity. This led to the analysis of 120 deceptive trials and 120 informative trials per participant. In addition, 16 out of the 30 participants underwent a session with a control condition consisting of an extra 160 control trials where no feedback was presented, here again with half the trials including introspective reports. To account for potential order effect, the control session was presented after one-third, two-thirds or at the end of the main experiment.

### Visual stimulation

Visual stimuli consisted of the superposition of two half transparent animated images, a face and a spiral, at the center of the screen (Iiyama ProLite E2483HS-B3). The spiral rotated around its center while the face alternatively opened and closed its mouth. Such superposition of half transparent animated streams has been shown to reduce the stability of the percept containing the two streams and thus facilitates the voluntary switch from one item to the other (Neisser and Becklen 1975; Clark 2017; Ransom *et al.* 2017). In addition, the two animated streams had to evoke distinguishable brain responses in order for our BCI to decode the attentional focus of the participant. We therefore continuously modulated the spatial phase scrambling of each item, eliciting “sweep” steady state visually evoked potential (ssVEP) responses (Ales *et al.* 2012; Norcia *et al.* 2015) at the frequency of 1.875 Hz for both streams but in temporal phase opposition.

To build our animated stimuli, we used 12 images of a face regularly spanning an animation of mouth opening and 8 images of a homemade spiral at different steps of a rotation animation. We cropped each image with a Gaussian filter to obtain smooth edges. We then inserted each image in a noisy background with the same Fourier amplitude. As a result, the items of both categories appear to emerge from the noise as phase scrambling decreases (Ales *et al.* 2012). Then, for each step of animation and each item we selected the correct amount of phase scrambling to produce the desired sweep ssVEP. Spatial phase scrambling was computed by a phase interpolation method (Ales *et al.* 2012). Finally, we superimposed pairs of images to create a complete dynamic stimulus of two superimposed animated images, producing oscillatory signals at the same frequency but in phase opposition.

In half of the trials, participants were asked to report the object of their decision (i.e. object they had decided to preferentially attend) and how confident they were about this decision. Both the object of decision and their confidence in that decision were reported at the same time on a four levels scale. Eight circles were thus displayed on a horizontal line (i.e. four circles for each object). A reference central dot was displayed between the two most central circles to ensure forced-choice decisions. Participants reported their decision by choosing to move the dot either to the left or to the right (counterbalanced across trials), and their confidence was rated by choosing a circle that was close (very unconfident) or far (very confident) from the reference central dot (see Fig. 1A).

### EEG recording

We recorded scalp EEG using a 64-channel Biosemi ActiveTwo system (Biosemi, Amsterdam, Netherlands). EEG analog signal was digitized at a 2048 Hz sampling rate. During recording, electrode offset was reduced to between ±50 μV for each individual electrode by softly abrading the underlying scalp with a blunt plastic needle and insulating the electrode tip with saline gel (Sigma Gel, Parker Laboratories, USA).

### Brain–computer interface

#### Overview

Our setup comprises one decoding computer and one stimulation computer. The stimulation computer continuously displays the visual stimulation (overlapping face and spiral) placed at the center of the screen oscillating in phase opposition. During the real-time experiment (practice, experimental and control phases), the decoding computer continuously receives EEG data and loads them in a buffer for on-line analysis (Oostenveld *et al.* 2011). Data are also saved for later offline analysis. During on-line analysis, the decoding computer outputs correlation scores for both items presented on the stimulation screen to the stimulation computer. At the end of each trial, the stimulation computer decides based on the correlation scores which stimulus has been preferentially attended during the decision phase (see Fig. 1B).

#### Decoding procedure

The decoding model was inspired by backward models of stimulus reconstruction used in recent psychoacoustics studies (O’Sullivan *et al.* 2015). The decoding computer continuously receives EEG data at a rate of 2048 Hz. Before further processing, the recorded data is down-sampled to 256 Hz. The EEG data is next filtered between 1 and 30 Hz with a one-pass Butterworth filter of order 6 and re-referenced to the average signal. Then from a 3 s segment R of EEG data, we try to infer a unidimensional signal *Ŷ* called the reconstructed signal that represents the visual stimulus most probably attended by the subject during this segment. Along the reconstructed signal, we also compute a representation of the two concurrent visual stimuli (the oscillation of the face and the oscillations of the spiral) called *Y_F_* and *Y_S_* (Andersen and Müller 2015). The reconstructed signal *Ŷ* and the abstract representations *Y_F_* and *Y_S_* of the visual stimuli are vectors with one value per time sample (256 samples/s in our case). The correlations scores *c_F_* and *c_S_* are obtained by correlating *Ŷ* and *Y_F_* on one hand and *Ŷ* and *Y_S_* on the other hand.


(1) $$c_{F}=\mathrm{corr}\;(\hat{Y},Y_{F})$$

(2) $$c_{S}=\mathrm{corr}\;(\hat{Y},Y_{S})$$

The stimulation computer thus receives one pair of correlation scores (*c_F_*, *c_S_*) every 250 ms computed over the three past seconds. Correlation scores were saved for offline analysis on the one hand and used to infer the preferentially attended item of the current trial on the other hand. At the end of the trial, we averaged the correlation scores over the last 1.5 s of the decision phase (6 correlations scores) for the face and spiral, respectively. The item having the higher averaged scores is designated as preferentially attended by the participants for this trial (see Fig. 1B).

Then the stimulation computer displays the appropriate feedback given the attended item (guessed by the model described beforehand) and the nature of the trial. This feedback corresponds to the decoded decision during the whole practice phase and for informative trials of the experimental phase. For deceptive trials of the experimental phase, the opposite item was displayed as feedback.

The reconstructed signal is obtained by applying a linear operation to the EEG data matrix R of dimensions time by channels. The model comprises a series of so-called lags *τ_k_* that account for how the stimulus experienced at time *t* influences the EEG data at time *t* + *τ_k_*. More precisely, we have


(3) $$\left(t\right)=\sum_{c,k}w_{ck}R\left(t+\tau k,c\right)$$

where *c* stands for channel and *k* for an index of our list of lags and *w_ck_* are the coefficients that define the backward model.

The model was trained from EEG data collected during the BCI training phase. Each trial was labeled with the item (face or spiral) the participants were asked to attend to during this trial. The EEG data were preprocessed prior to the training of the model by applying a common average reference (mean EEG is subtracted from all channels) and filtering between 1 and 30 Hz with the same one pass Butterworth filter of order 6 that we use for online decoding procedure.

We find the coefficients *w_ck_* of the backward model by solving the regression problem


(Equation 4) Y=wX

where the regressor variable *X* contains EEG data from all trials and channels and *Y* contains the representation of the attended stimulus at each trial (see details in O’Sullivan *et al.* 2015). To evaluate the accuracy of this model, we used a cross-validation procedure on all trials from the BCI training phase. The decoding accuracy of the model reached 78.1% (*SD* = 11.6%). The results of this cross-validation procedure for each individual are shown in Supplementary Fig. S1.

### Experimental procedure

The experimental protocol was divided in three phases: participants first underwent the BCI training and the participants’ practice phases before performing the main phase. The main phase consisted of three identical blocks to which we added a 4th control block for 16 participants.

#### BCI training phase

To allow our BCI to decode the preferentially attended item in real time, we first gathered labeled data to train the participant’s individual model. At the beginning of each of the 30 trials of this phase, a target was designated by a letter (F for face, S for spiral) overlapping a fixation cross at the center of the screen for 1 s. Participants were asked to preferentially attend the designated items during the whole 5 s of the trial.

Instructions were given as follows: “You will see two superimposed items, a face and a spiral. At the beginning of each trial one of them will be designated as the current target. We will ask you to focus as much as you can on the target for the 5 s during which the image is displayed on the screen. Please refrain any eye movement while the image is displayed”.

#### Participants’ practice phase

Participants were then offered to familiarize with the BCI setup for 20 training trials. Further details on this phase can be found in the Supplementary data.

#### Main phase

Participants then performed 480 trials presented in three successive blocks of 160 trials of 5 s. Visual stimulation was continuously displayed on the screen across trials and disappeared only every eight trials. No target was designated and participants were encouraged to choose their object of attention (i.e. face or spiral) and to change at will across trials. As for the practice phase, feedback was provided for 500 ms at the end of each trial about the object participants preferentially attended. Crucially, feedback was only informative (reflecting the allegedly attended item) in 75% of the trials (Informative trials). In the remaining 25% of the trials, the other item was displayed instead (Deceptive trials). During one-third of the informative trials and during all deceptive trials, participants were asked to report the object they decided to attend, and to perform a confidence judgment about this decision (very unconfident, unconfident, confident, very confident). This distribution of report requests provides an equal amount of report following informative and deceptive trials for our analysis. Furthermore, it ensures that receiving a report request does not inform participants on whether the feedback was informative or deceptive.

Instructions were given as follows: “In this phase you will not be asked to attend a specific target. Please decide which one you would like to focus on. You can switch to the alternative item whenever you feel like doing it. Please refrain from adopting a specific strategy or trying to create a sequence. Regularly, feedback about your decision will be displayed, as for the previous phase. Moreover, you will sometimes be asked to report what object you were attending at the moment and to rate your confidence about this report. Importantly, the feedback you receive can sometimes be deceptive, not reflecting what you were focusing on. Finally, please restrain your eye movement to period without stimulus on screen or when you are reporting your decision.”

#### Control phase

Sixteen participants performed one block of 160 trials for the control phase. This phase is the same as in the Main phase except that no feedback was provided at the end of the trials. The control block and the three blocks of the main phase were presented in random order counterbalanced across participants.

Instructions were given as follows: “This block is the same as the previous one except that you will not receive any feedback regarding your recent decision.”

### Data processing

#### Internal decision evidence

For each trial, we compute a correlate of participants’ internal evidence supporting their recent decision. During the decision phase, our BCI outputs every 250 ms correlation scores associated with each object computed over 3 s long window (Fig. 1B). These correlation scores reflect how close are the brain signals from being generated by the observation of the face or the spiral respectively. Then, our proxy for internal (decision) evidence (IE) consists in the absolute value of the accumulated difference between the correlation scores associated with each object over the last 1.5 s of the decision phase (including 4.5 s of data in total), and measures how EEG signals separate the two items over the 4.5 s before the feedback apparition.

#### Accuracy of introspective reports

We determined for every trial the preferentially attended item the same way we did it online (see Decoding procedure in brain–computer interface section of the Methods). We operationalized introspective accuracy as being correct when the reported object matches the object decoded by the BCI (e.g. for instance face reported, face decoded), and incorrect otherwise (e.g. face reported, spiral decoded).

#### Confidence in introspective report

At the end of the trials, participants reported the confidence they have in their decision along with the decision itself on a 2 × 4 points scale. Confidence reports were median-split, reports of confidence 1 and 2 were labeled as “Low confidence” trials and reports of 3 and 4 were labeled as “High confidence”. For modeling purposes, confidence was coded as a 2-level factor.

#### Consistency of internal decision evidence

We computed an index approximating for each trial the ratio of the internal decision evidence strength over its variance. Our consistency index is thus described by the formula:


$$\mathrm{Consistency}=\left|\frac{\mathrm{Internal}\;\mathrm{Evidence}}{\mathrm{Var}\left(\mathrm{Correlation}\;\mathrm{difference}\right)}\right|$$

where Internal Evidence is described in the previous paragraph and Var(Correlation difference) represents the bootstrapped variance of the correlation difference over the period of accumulation (3 s before the feedback apparition).

### Statistical analysis

Summary statistics were calculated in Matlab (Matlab 2018, The MathWorks, Inc.). All other statistical tests were calculated in R [R version 3.6.0 (Team 2012)]. Before applying pairwise comparison, the Shapiro–Wilk method was used to test for the normality of the data. If the normality hypothesis was not rejected, we applied a two-sided paired Student’s *t*-test to our data. Data containing too many empty values or not meeting normality assumptions were analyzed with Wilcoxon rank test. Holm–Bonferroni corrections for multiple comparisons were calculated with R. Cohen *d* was calculated using the R effsize library (Torchiano 2016) for approximating effect size.

Both linear mixed effect models and generalized linear mixed effect models (GLMEs) were fitted using lmer4 packages (Bates *et al*. 2015). Cumulative linked mixed models were fitted using ordinal package (Christensen 2019) and using the full (4 points) confidence scale. To operate model reduction we removed non or least significant terms and compared Akaike information criterion of more complex and simplified models. Moreover, we ran a Chi-square test to decide whether the more complex model was significantly better at explaining our data. The reported *P*-values of each fixed effect of linear mixed-effects models and generalized linear mixed-effects models were obtained with this Chi-square test comparing one model with all the possible simplification obtained by removing a single effect. For each model, we detailed the model reduction procedure (see Supplementary Materials).

## Results

### Impact of internal decision evidence and external cues on introspection

Does introspection integrate internal evidence supporting just-made decisions or is it a pure reconstructive process shaped by external cueing? We here operationalize the report accuracy as being correct when the reported object matches the object decoded by the BCI (e.g. for instance face reported, face decoded), and incorrect otherwise (e.g. face reported, spiral decoded). Together with this measure of report accuracy, we computed a proxy for internal evidence (IE) supporting the recent decision. IE measures how strongly participants preferentially attended one object over the other one (see Methods). Finally, the type of feedback cue displayed on each trial was encoded as either deceptive (opposite to IE in 25% of the trials) or informative (corroborating the IE in 75% of the trials). In the case of a deceptive feedback, a correct report corresponds to a genuine introspection while an incorrect report corresponds to a confabulation. However, in the case of informative trials, reports accuracy does not inform on the nature of introspective mechanisms because participants can be correct just by blindly accepting the feedback (see Supplementary Results for more details). To determine the respective influences of internal decision evidence and external information on introspective processes, we modeled accuracy using IE and the type of feedback cue as fixed effect and participants as random effect.

**Figure 2.**
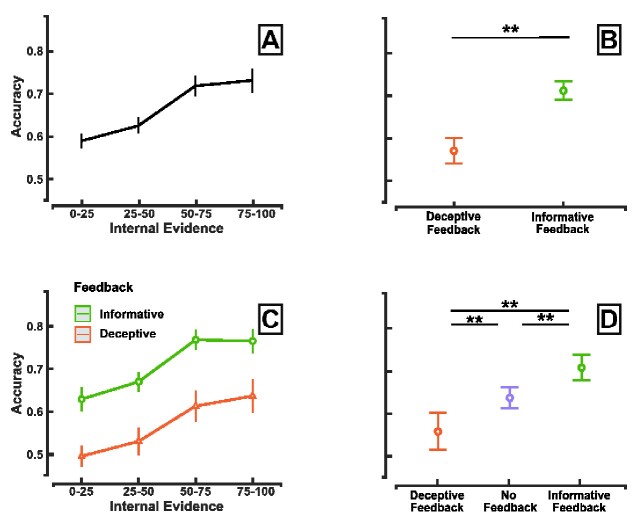
Respective influences of internal decision evidence and external cues on introspective reports. (**A**–**C**) Impact of internal evidence (IE) on the accuracy of introspective reports. For each participant, we computed the distribution of IE across trials in terms of percentile. Vertical bars represent bootstrapped confidence intervals across participants (1000 iterations). (**B**–**D**) Impact of the feedback cue on the accuracy of introspective reports. Error bars represent the standard error to the mean (SEM). *N* = 30 in A, B and C. *N* = 16 in D. Significance marker: ** p<0.01.

We first thought to determine the relationship between the internal evidence that was available during the decision phase and the accuracy of introspective reports. As shown in Fig. 2A, report accuracy significantly increases with the amount of internal evidence [GLME: odds ratio (OR) = 1.33, confidence interval (CI) (1.26–1.41), *χ*^2^ = 211.5, *P* < 0.001]. Yet, an alternative hypothesis would be that trials with high IE are less subject to decision misclassification by the BCI. In such case, participants would systematically correctly report their recent decision, yet, classification and thereby accuracy will be higher for trials showing high IE. To account for this alternative interpretation, we compared the internal evidence in correct and incorrect trials during the practice phase. In this phase, participants were assigned to focus on a given item such that correct trials could be interpreted as BCI correct classification and incorrect trial as a misclassification. Results are shown in Supplementary Fig. S7 and confirm that IE is not affected by the accuracy of our BCI classification [LMER: estimate = 0, CI (−0.25 to 0.26), *χ*^2^ = 0.00, *P* > 0.9]. Indeed, internal evidence did not differ between correct and incorrect classification (pairwise comparison: *z* = −0.39, *P* > 0.5), ruling out the possibility that an IE reflects classification performance.

Furthermore, the type of feedback had a significant influence on introspective reports as we observed a higher accuracy following an informative feedback (*M* = 0.71, *SD* = 0.11), compared with a deceptive feedback (*M* = 0.57, *SD* = 0.16) (Fig. 2B, more detail on Supplementary Fig. S3 and Supplementary Table S1) [OR = 1.72, CI (1.45–2.04), *χ*^2^ = 151.2, *P* < 0.001], revealing that the type of contextual cue influences introspective reports. Importantly, the nature of the feedback could not influence the accuracy of the BCI classifier. Indeed, the nature of the feedback was assigned for each trial before the beginning of the experiment and independently of the on-line classification process. Moreover, we found no significant interaction between IE and the type of feedback [OR = 1.04, CI (0.98–1.17), *χ*^2^ = 2.2, *P* > 0.1], revealing that feedback cues modulate the accuracy of introspective reports regardless of the internal information available during the decision phase (Fig. 2C).

Are introspective reports modulated by both deceptive and informative feedback cues? To address this question, we compared the effect of each type of feedback to a baseline condition where no feedback was provided. Sixteen out of the 30 participants performed an additional block with the exact same structure except that no feedback cue was presented between the decision and report phase. Nature of the feedback impact participants’ report accuracy (GLMER *χ*^2^ =86.1, *P* < 0.001, Supplementary Table S2). The results of this control session confirm that informative feedback presentation increases the accuracy of participants’ reports compared with reports without feedback [GLMER, OR = 1.45, CI (1.24–1.70), *χ*^2^ = 21.3, *P* < 0.001, Supplementary Table S3]. Conversely, we observed that accuracy decreases following a deceptive feedback compared to a condition without feedback [GLMER, OR = 0.74, CI (0.63–0.86), *χ*^2^ = 15.1, *P* < 0.001, Supplementary Table S4, see Fig. 2D, Supplementary Fig. S3B and Results]. Together, these results reveal that both internal decision evidence and external cues influence participants’ introspective reports.

### Metacognitive failures

We then studied whether external cueing impacts not only reports, but also decision confidence. Confidence is known to track performance for decisions conducted under perceptual uncertainty (Yeung and Summerfield 2012; Pouget *et al.* 2016). Therefore, we aimed at investigating the relationship between introspection and confidence, and in particular whether the misleading influence of the deceptive feedback would also impact the confidence associated with introspective reports. We thus modeled introspection accuracy using confidence and the type of the feedback as fixed effect and participants as random effect.

**Figure 3.**
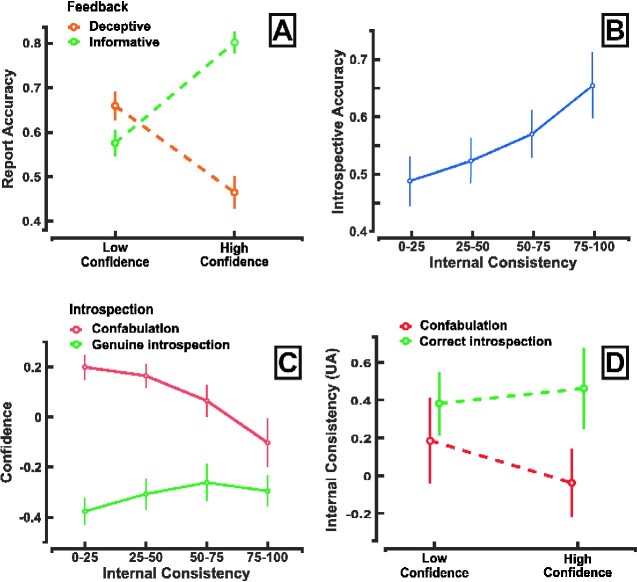
Confabulations are associated with higher confidence when decisions are supported by inconsistent internal evidence. (**A**) Effect of feedback on the accuracy–confidence relationship. Accuracy in *y*-axis is the percentage of correct trials and was computer for trial associated with respectively a low and a high confidence separately for deceptive (orange) and informative (green) feedback. Vertical bars represent bootstrapped confidence intervals across participants (1000 iterations). (**B**) Effect of internal consistency on introspective accuracy. For each participant, we computed the distribution of internal evidence across trials in terms of percentile. Accuracy was then computed within each quartile and participants. Vertical bars represent standard error to the mean. (**C**) Effect of internal consistency on confidence in confabulation and genuine introspection. Confidence was rated on a 4 points scale (from 1 for very unconfident to 4 for very confident). For each trial, we normalize the confidence rating by subtracting the participant’s confidence rating average to each trial rating. For each participant, we computed the distribution of internal evidence across trials in terms of percentile. Confidence was then computed within each quartile and participants. Vertical bars represent standard error to the mean. (**D**) Internal decision evidence consistency given confidence in confabulation and genuine introspective reports. Internal evidence consistency in *y*-axis was averaged across trials grouped by confidence and accuracy. Only trials followed by deceptive feedback are represented as this condition allows to disentangle correct introspection from confabulation.

We found that when participants are presented with a deceptive feedback cue, the classical relationship between confidence and accuracy is overturned. We observed a significant interaction between the nature of the feedback and the confidence attributed to decision reports [GLME: OR = 3.46, CI (2.29–4.01), *χ*^2^ = 274.4, *P* < 0.001, Supplementary Table S5] (Fig. 3A). Indeed, when participants received an informative feedback, we found a positive correlation between confidence and report accuracy (high confidence: *M* = 0.80, *SEM* = 0.02; low confidence: *M* = 0.58, *SEM* = 0.03, z = 5.2, *d* = 1.53, *P* < 0.0001 signed-rank test). However, strikingly, when participants received a deceptive feedback, this correlation was inverted, with confidence rising up as accuracy decreased (high confidence: *M* = 0.46, *SEM* = 0.04; low confidence: *M* = 0.66, *SEM* = 0.03, *z* = −4.19, *d* = −1.07, *P* < 0.0001 signed-rank test). Moreover, we found that participants exhibit a lower confidence after genuine introspection (*M* = 1.39 *SEM* = 0.04) than for their confabulations (*M* = 1.58, *SEM* = 0.04) (signed rank test, z = 4.6, *d* = 0.93, *P* < 0.0001, see Supplementary Fig. S5). Together, these results reveal that a deceptive feedback can not only delude participants about the choices they made (i.e. CB) but also falsify the feeling of confidence they associated with their introspection (i.e. aberrant metacognitive failures).

### Reliability of internal decision evidence

How can external cues impact qualitative aspect of introspection such as the confidence? To better understand the underlying mechanisms of this overconfident confabulations, we investigated the conditions that permit external cues to prevail during introspective reports. The human brain constantly integrates information coming from multiple noisy sources. Several models propose that in multi-sensory perception, the respective participation of each source of evidence to the final percept is regulated by their own strength and reliability (Ernst and Banks 2002; Knill and Pouget 2004; Moore and Fletcher 2012). Here, we propose that a similar mechanism operates between internal decision evidence and external cues in the production of introspective reports. Since external cues are kept constant, introspective processes should be dominated by external cues if the internal decision evidence are inconsistent (i.e. weak and noisy).

We built an index of internal consistency accounting for the strength and reliability of internal decision evidence during each trial. To compute internal consistency, we took for each trial the ratio of the internal decision evidence strength divided by its variance (see Methods). Then, to understand how internal decision consistency evolves during confident confabulations, we modeled internal consistency using accuracy and confidence as fixed effect and participants as random effect. To distinguish confabulations from genuine introspection, this analysis was restricted to deceptive trials (see Supplementary Results). We predicted that confabulations associated with high confidence correspond to decisions supported by low internal consistency. In such cases, external cues should prevail in the formation of introspective reports, both in the reported decision and in the associated confidence.

First, participants’ introspective accuracy increases with internal consistency [GLMER, OR = 1.25, CI (1.17–1.32), *χ*^2^ = 56.5, *P* < 0.001, see Supplementary Table S6 and Fig. 3B]. In other terms, when internal variables are weak and noisy, participants’ introspective reports tend to reflect external cues rather than their recent decision. On the other hand, when internal variables are consistent, participants’ reports reflect their original decision despite deceiving external cues.

Furthermore, to assess the effect of internal consistency on confidence for genuine introspection and confabulations, we regress confidence using consistency and introspective accuracy as predictors. Confidence in genuine introspection increases with internal variables consistency (Fig. 3C) [CLMM, OR = 1.09, CI (1.02–1.17), *χ*^2^ = 6.1, *P* < 0.05, Supplementary Table S8]. However, this relationship was inverted for confabulation [CLMM, OR = 1.20, CI (1.09–1.33), *χ*^2^ = 13.5, *P* < 0.001, Supplementary Table S7] since confidence decreases with internal consistency [CLMM, OR = 0.9, CI (0.84–0.97), *χ*^2^ = 8.4, *P* < 0.01, Supplementary Table S9].

Consequently, as shown in Fig. 3D, confabulations with high confidence show markedly lower internal consistency compare with confabulation with low confidence [LMER: estimate = −0.11, CI (−0.21 to −0.01), *χ*^2^ = 4.9, *P* < 0.05, Supplementary Tables S10–S12] (low confidence: *M* = 0.15, *SEM* = 0.07; high confidence *M* = −0.08, *SEM* = 0.05), signed rank test *z* (29) =2.4, *d* = 0.51, *P* < 0.05. On the other side during genuine introspection, higher confidence tend to be supported by higher internal consistency [LMER: estimate = 0.07, CI (−0.01 to 0.15), *χ*^2^ = 2.9, *P* = 0.09, Supplementary Table S13] (low confidence: *M* = 0.35 *SEM* = 0.05; high confidence: *M* = 0.43, *SEM* = 0.06; paired *t*-test *t* (29) = −1.32, *d* = −0.19, *P* = 0.19).

Altogether, these results reveal that confabulation occurs when internal decision evidence are weak and noisy. Furthermore in such condition, external cues influence not only the content but also the metacognitive aspects of introspective reports on decision.

## Discussion

In the present study, we used a BCI to covertly track a correlate of internal evidence supporting decisions and study how it affects introspective illusions. We found that participants’ introspective reports combine both internal decision evidence and external cues about their decisions. When presented with feedback cues that opposed their original internal decision evidence, participants tended to report it as reflecting their own decision. Furthermore, we found that the noisier and weaker was the original internal decision evidence, the more they were confident about having made the choice corresponding to the external cue. In other words, external cues dominate introspective reports and decision confidence when internal evidence supporting the original decision was inconsistent.

Combining multiple sources of noisy information has been proposed to account for multisensory perception (Ernst and Banks 2002; Knill and Pouget 2004) and for the sense of agency (Synofzik *et al.* 2008; Moore and Fletcher 2012; Legaspi and Toyoizumi 2019). The sense of agency appears to result from an integration of both internal motor signals (Blakemore *et al.* 2002; Haggard and Clark, 2003; Haggard, 2017) and external information (e.g. action outcome) (Moore and Haggard 2008; Moore *et al.* 2009). Moreover, this integration appears to follow Bayesian principles, whereby sources of information are weighted by their respective reliability (Moore and Fletcher 2012; Legaspi and Toyoizumi 2019). Here, we propose to extend this framework to account for introspective illusions such as CB. In the context of our study, both external cues and internal variables (here indexed by a correlate of internal evidence supporting the decision) can be considered as noisy sources of information with their own relative contributions to introspection. Therefore, we propose that external cues are combined with internal decision evidence in inverse proportion to internal evidence availability and reliability when forming introspective reports (for similar accounts in the perceptual domain, see Ernst and Banks 2002; Clark and Yuille 2013; Farrell and Lewandowsky 2018). That is, when a choice was made on the basis of weak or unreliable internal evidence, introspective reports are more likely to be dominated by exogenous elements such as the decision’s outcome feedback, thereby resulting in a CB episode.

To unravel which factors influence CB, previous studies relied on behavioral measures and compared the detection rate of deceptive trials across various types of stimuli. For instance, decisions involving familiar choices (e.g. known brands, political preferences, etc.) are rarely followed by CB episodes (Hall *et al.* 2010; Hall *et al.* 2012; Sauerland *et al.* 2014; Somerville and McGowan 2016; Rieznik *et al.* 2017; Strandberg *et al.* 2018). Our work offers an interpretation of those findings by suggesting that familiarity with the choices might increase the weight of internal decision evidence during introspection. Consequently, the influence of internal decision evidence on introspection will prevail over the influence of deceptive cues, thus improving introspective accuracy.

Conversely, one might expect a similar effect if, instead of increasing the consistency of internal decision evidence, it was the external outcome consistency that was decreased. For instance, a recent study (Reyes *et al.* 2018) manipulated the confidence that participants have on the experimenter and showed that they undergo stronger CB effects when the experimenter appears in control of the experimental setup or if they have been primed about her professionalism. On the other hand, when primed with an apparent lack of competence of the experimenter or if the experimenter looks overwhelmed by a fake bug on the experimental setup, the detection of deceptive trials largely increases.

Other attempts to address the underlying mechanisms of CB phenomena relied on linguistic analysis but failed to differentiate between reports following deceptive versus non-deceptive trials (Johansson *et al.* 2005, 2006; Johansson *et al.* 2008). Together with previous studies (Nisbett and Wilson 1977), these results argue that introspective reports are based on participants’ belief about their decision rather than the mental states supporting those decisions. Participants remain ignorant of those underlying mental states even in the absence of deceptive feedback (Petitmengin *et al.* 2013). Our results corroborate those conclusions by offering a mechanistic account for why no linguistic difference should be observed between reports following deceptive and non-deceptive feedback. Indeed in both types of trial, internal evidence supporting the decision can be weak and noisy, leading subsequent justifications to mostly reflect the feedback presentation rather than internal variables. Therefore, no difference should be expected in the justification of informative and deceptive trials.

While the use of confidence judgment is widespread in psychological studies, its relationship with confabulation is still unclear. While some argue that introspective illusions are subjectively indistinguishable from genuine introspection (Carruthers 2009, 2010), other studies show that illusions often come with a reduced confidence (Nisbett and Wilson 1977; Wheatley and Haidt 2005; Hall *et al.* 2012; Rieznik *et al.* 2017; Strandberg *et al.* 2018). Altogether, our results nuance this debate by showing that the subjective distinction between confabulation and genuine introspection depends on the availability and reliability of internal variables. When internal decision variables are weak and noisy, confabulation can’t be distinguished from genuine introspection, and both will be reported with high confidence (Carruthers 2009, 2010). If the consistency of the internal decision evidence is high, participants directly access their recent decision variables and easily detect external manipulations. Finally, if the internal evidence supporting decisions shows intermediate consistency, participants will eventually fail to notice external manipulation but their subjective experience will be affected as they report a lower confidence compared to genuine introspection (Fiala and Nichols 2009) (see Fig. 3B).

Although our task presents many similarities with the original CB paradigm, we must note that it also differs on several aspects. We manipulate the feedback more often (random ordering assignment in 25% of the trials) and in a more explicit manner (participants were informed of the potential deceptive nature of the outcome) compared with the original paradigm. Importantly, however, we still observed a large portion of CB episodes though reduced compared to the original study (40% here versus 60–80% in original study) (Johansson *et al.* 2005) (see Fig. 2B and Supplementary Fig. S3A).

In the present study, we propose that participants could introspect on some of their internal information but remain subject to introspective illusions when those information are weak and noisy. Yet this interpretation relies on the assumption that our BCI decode accurately the object participants choose to focus on. Nonetheless, our BCI could sometimes misclassify participants decision, leading to the presentation of the opposite feedback. The reliability of our decoder approximated 78% during externally driven choice (i.e. the BCI training phase). In line with recent findings (Schurger *et al.* 2012; Murakami *et al.* 2014; Wisniewski *et al.* 2016; Brass *et al.* 2019), performance of the decoder should comparable during the main experiment. Yet, the condition of the decision differed between BCI training phase and the main experiment. If participants were assigned to focus for 5 s on a given item in the BCI training phase, they were free to switch at will in the latter condition. Therefore, our decoding method could be sensitive to decision changes in the last second of the decision phase. Moreover, we checked that the modulation of accuracy that we attributed to IE or internal consistency did not reflect instead variation in the BCI decoding performance (see Supplementary Results and Figs S6 and S7). In addition, to account for the potential misclassification due to late change of decision, we first identified trials where those changes potentially occurred and confirmed our results after having removed them (see Supplementary Materials).

## Conclusion

In conclusion, we combined a CB paradigm with a BCI to demonstrate that introspective reports about recent, private decisions result from the integration of internal evidence and external cues. When internal variables supporting the original choice are weak and noisy, participants accept external outcomes as their original intention even when the two are in contradiction. Moreover, our study reveals that not only the object of a decision but also the metacognitive aspects of this decision are subject to reconstruction. When internal decision evidence is weak or unreliable, participants show high confidence for their confabulations. Our study shed new lights on the mechanisms underlying introspective illusions, unraveling a continuum in the awareness people have about their decisions. Indeed, their introspective experience ranges from relying on internal information to being purely driven by external factors as a function of the availability and reliability of the evidence supporting their original decision.

## Supplementary data

Supplementary data is available at *NCONSC Journal* online.

## Supplementary Material

niab004_Supplementary_Data
